# Examining Movement Patterns, Skeletal Muscle Mass, and Hip Mobility in Office Workers With or Without Lower Back Pain: An Analytical Cross-Sectional Study

**DOI:** 10.7759/cureus.64721

**Published:** 2024-07-17

**Authors:** Ryo Miyachi, Yoshinari Fujii, Takaaki Nishimura, Akio Goda, Yui Nagamori, Yuji Kanazawa

**Affiliations:** 1 Physical Therapy, Faculty of Health and Medical Sciences, Hokuriku University, Kanazawa, JPN; 2 Internal Medicine, Faculty of Health and Medical Sciences, Hokuriku University, Kanazawa, JPN; 3 Physical Medicine and Rehabilitation, Faculty of Health and Medical Sciences, Hokuriku University, Kanazawa, JPN; 4 Neurology, Faculty of Health and Medical Sciences, Hokuriku University, Kanazawa, JPN; 5 Anatomy, Faculty of Health and Medical Sciences, Hokuriku University, Kanazawa, JPN

**Keywords:** hip mobility, muscle mass, office worker, movement pattern, lower back pain

## Abstract

Objectives: The purpose of this study was to clarify the relationship between Functional Movement Screen (FMS), skeletal muscle mass, and hip mobility in office workers with or without chronic lower back pain (LBP), as well as to determine whether the above items differed between office workers with or without chronic LBP.

Methods: This study utilized an analytic cross-sectional design. The participants were 35 office workers (14 in the LBP group and 21 in the non-lower back pain group, or NLBP) who were willing to cooperate with the request for cooperation in this study. Movement patterns were assessed by FMS and skeletal muscle mass was measured by bioelectrical impedance analysis. Hip mobility was measured by prone hip extension (PHE) and straight leg raising. The correlations between each item and differences in the presence or absence of LBP were analyzed.

Results: The LBP and NLBP groups showed different correlations (p<0.05) between total and subcategory scores and skeletal muscle mass. Total FMS score (p=0.02, r=-0.40) and PHE angle (p=0.01, r=0.43) were significantly higher in the LBP group than in the NLBP group.

Conclusions: The FMS shows different relationships between total and subcategory scores and skeletal muscle mass for office workers with or without LBP. In addition, office workers with LBP may have different movement patterns and greater hip extension range of motion than those without LBP.

## Introduction

Office workers who spend most working hours sitting often experience lower back pain (LBP). LBP has a lifetime prevalence rate of 84% and is one of the most common health problems in modern society [1,2]. In recent years, changes in the working environment have increased the demand for prolonged sitting [3], and an increasing number of reports have suggested a relationship between prolonged sitting and LBP [4,5]. LBP is a serious issue among office workers because it not only affects the quality of life but also decreases productivity and increases absenteeism at work [1,6].

In the management of LBP, it is important to evaluate not only the lumbar spine but also the whole body. For example, decreased mobility of the shoulder joint and thoracic spine leads to a compensatory increase in lumbar motion and dysfunction of the lumbar spine [7]. Regarding the lumbar spine and lower limb joints, such as the hip and knee joints, dysfunction of one joint can also affect the other, leading to tissue damage [8-11]. Thus, the reciprocity of movements is seen not only in adjacent joints but also in the entire body. In particular, office workers are susceptible to decreased mobility and muscle endurance in certain areas because they hold the lower extremities and trunk in flexed positions for long periods [12], and dysfunctions originating from other areas may also affect the lumbar region. Therefore, to treat LBP, an approach that considers not only the lumbar region but also the whole-body connection is required.

From this perspective, the Functional Movement Screen (FMS) is widely used to assess and score the quality of movement (movement patterns) of the whole body. Originally, the FMS was a test battery developed for injury prevention in athletes [13,14], but recently, it has been utilized for a variety of people, including the elderly, children, and those with pain [15-17]. The FMS is often used to assess whole-body movement patterns [15,18].

FMS consists of seven items that are subcategorized into primitive movement patterns and higher-level movement patterns (deep squat, hurdle step, and in-line lunge) [19]. Primitive movement patterns are divided into basic mobility and stability movement patterns (shoulder mobility and active straight leg raise, or ASLR) and transitional movement patterns (trunk stability pushup and rotary stability) that require stability, coordination, and control [19]. These subcategories provide important clues for interpreting body weaknesses and performing corrective exercises. However, none of the FMS assessments of movement patterns, including the subcategories, have examined their relationship with other factors, such as skeletal muscle mass and hip mobility, which may be associated with LBP [20,21], and have not compared movement patterns between LBP and non-LBP participants.

Therefore, the purpose of this study was to investigate the relationship between FMS scores, including subcategories, skeletal muscle mass, and hip mobility, in office workers with or without chronic LBP and to determine whether there were differences in the above items between office workers with or without chronic LBP.

## Materials and methods

Study design

This study was an analytic cross-sectional design, and measurements were taken at the laboratory of Hokuriku University between February 2023 and May 2023. Office workers who were willing to participate in the study were included. The study considered those who spent more than half of their working time in a sitting position, had worked in their current job for at least one year, and were working full-time.

The exclusion criteria were as follows: (1) typical physical disabilities such as paralysis or cognitive dysfunction, (2) implants or other metal objects such as pacemakers inserted into the body, (3) a history of surgery within the past 12 months, and (4) pregnancy. Information on LBP (Numerical Rating Scale, Oswestry disability index, and duration) was obtained in a preliminary survey.

Those with pain localized to the lumbar region lasting more than three months were defined as the LBP group and those without LBP pain as the NLBP group. Those who did not fall into either category (e.g., acute LBP) were excluded from the study, as were those with neurological symptoms in the lower extremities. None of the participants in the NLBP group had a history of LBP or other back disorders. For all measurements, the raters did not know whether the participants belonged to the LBP or the NLBP group. The sample size was calculated using G*Power v.3.1. (Heinrich-Heine-Universität Düsseldorf, Düsseldorf, Germany) with an effect size of 0.4, an alpha of 0.05, and a power of 0.8, resulting in 34 participants.

Ethical considerations

This study was conducted in accordance with the principles of the Declaration of Helsinki. The purpose and content of the study, the fact that the obtained data would not be used for any purpose other than the study, and precautions against the leakage of personal information were fully explained to the participants in advance. Consent to participate in the study was obtained from all the participants after obtaining their signatures. This study was approved by the Ethics Committee of Hokuriku University (approval number: 2023-2).

Measurements

The FMS is scored on a 4-point scale from 0 to 3 for each item. Scoring was given as 0 points if there was pain, 1 point if the test could not be completed, 2 points if the task could be completed but some compensation was required to perform the task, and 3 points if the task could be performed correctly without any compensation [13]. The FMS used in this study was modified to apply to the LBP population, giving a score of 0 only if there was an increase in LBP during measurement [15]. For items with two sides during the evaluation, the value of the side with the lower score was recorded, and the FMS score was calculated. The final score on the FMS-FS was the sum of all FMS test scores; the higher-level movement pattern item score on the FMS-HS was the sum of the higher-level movement pattern test scores; the basic mobility and stability movement pattern item score on the FMS-BS was the sum of the basic mobility and stability movement pattern test scores; and the transitional movement pattern item score on the FMS-TS was the sum of the transitional movement pattern test scores (Table 1). For the ASLR, the angle measurements described below were conducted simultaneously with FMS scoring. FMS was measured using the FMS test kit (FMS kits, Functional Movement System, USA) by a physical therapist who was FMS-certified and had at least five years of experience in measuring FMS.

**Table 1:** Functional Movement Screen subcategories and constituent tests

Subcategories		Tests
Primitive movement patterns	Basic mobility and stability movement patterns	Shoulder mobility
		Active straight leg raises
	Transitional movement patterns	Trunk stability pushup
		Rotary stability
Higher-level movement pattern		Deep squat
		Hurdle step
		In-line lunge

Skeletal muscle mass was used as a measure of muscle mass in this study because it is a whole-body segment assessment index that is independent of the direction of movement and is not affected by fatigue or psychological factors. Skeletal muscle mass was measured by bioelectrical impedance analysis using a body component analyzer (Inbody 270, Inbody Japan Inc., Tokyo, Japan). Bioelectrical impedance analysis has been utilized as a non-invasive, simple, and safe alternative to dual-energy X-ray absorptiometry [22]. Measurements were taken in the standing position with bare feet on the foot sensor of a body component analyzer. After measuring the body weight, the hand sensor was grasped. The skeletal muscle masses of the upper and lower limbs, trunk, and skeletal muscle index (SMI) were used as indices of skeletal muscle mass. The average muscle masses of the upper and lower limbs were used as representative values for the left and right sides, respectively.

Hip mobility was measured by capturing the prone hip extension (PHE) and ASLR angles using a fixed camera. The camera was positioned 2 meters lateral to the participant’s hip joint at a height of 30 cm. Both tasks were performed with active motion and the participants stopped at the end of the task. Participants were instructed to perform movements as wide as possible. For the PHE, one co-author had the participants perform the task with their pelvis immobilized to prevent pelvic movements. From the obtained videos, the tilt angle of the thigh to the floor at the end position of the task movement was measured using the image analysis software ImageJ (Wayne Rasband, National Institutes of Health (NIH), Bethesda, USA). The intraclass correlation coefficient (ICC (1,1)) was measured for 10 participants in contrast to the LBP and NLBP groups in this study, and it was confirmed that the measurement reliability was sufficient (ASLR: 0.98, PHE: 0.90). Average left and right hip angles in the PHE and ASLR groups were used as representative values.

Statistical analysis

Statistical Package for the Social Sciences (IBM SPSS Statistics for Windows, IBM Corp., Version 28.0, Armonk, NY) was used for statistical analysis. Because the FMS is an ordinal scale, nonparametric tests were used to test for it. The Shapiro-Wilk test was used to test for normality. Spearman's correlation analysis was used to evaluate the relationships between each item. For the differences between the LBP and NLBP groups, a chi-square test was used to compare the proportions of those who had a left-right difference in the FMS items, those who had a score of 1, and those who had a score reduction in each FMS item that included the word "spine" or "torso" as a criterion, as well as the proportions of male and female LBP and NLBP groups. The Mann-Whitney U test was used to compare FMS scores, and unpaired t-tests were used for age, height, weight, skeletal muscle mass, and hip mobility. The significance level was set at p < 0.05.

## Results

Thirty-five participants (20 males and 15 females, age of 45.5 ± 9.9 years, height of 165.6 ± 9.0 cm, and weight of 62.4 ± 11.4 kg) were included in the study (Table 2). The measured values of each item are listed in Table 3. None of the participants scored 0 on the FMS scale.

**Table 2:** General participant characteristics Values are presented as number of participants (percentage) or mean (standard deviation). LBP: lower back pain; NLBP: non-lower back pain; NRS: numerical rating scale; ODI: Oswestry Disability Index

Characteristics	Overall (N=35)	LBP group (N=14)	NLBP group (N=21)	p-value
Sex (N)	Male: 20 (57.1); Female: 15 (42.9)	Male: 8 (57.1); Female: 6 (42.9)	Male: 12 (57.1); Female: 9 (42.9)	>0.99
Age (years)	45.5 (9.9)	42.7 (7.9)	47.4 (10.8)	0.18
Height (cm)	165.6 (9.0)	167.4 (8.9)	164.4 (9.0)	0.33
Weight (kg)	62.4 (11.4)	64.9 (10.8)	60.8 (11.7)	0.31
Daily sitting time at work (h)	7.1 (1.9)	6.9 (1.7)	7.3 (2.0)	0.47
NRS (score)	-	3.5 (1.6)	0	-
ODI (score)	-	5.9 (3.3)	0	-

**Table 3:** Differences in each measure between the lower back pain and non-lower back pain groups Values are presented as median (interquartile range) or mean (standard deviation). * Significant difference between groups (p < 0.05). LBP: lower back pain; NLBP: non-lower back pain; CI: confidence interval; FMS-FS: final score on the Functional Movement Screen; FMS-HS: higher-level movement pattern item score on the Functional Movement Screen; FMS-BS: basic mobility and stability movement pattern item scores on the Functional Movement Screen; FMS-TS: transitional movement pattern item score on the Functional Movement Screen; PHE: prone hip extension; ASLR: active straight leg raise; SMI: skeletal muscle mass index

	Overall (N= 35)	LBP group (N=14)	NLBP group (N= 21)	p-value	Effect size (r)
FMS-FS (score)	14.0 (11.0 to 15.0)	15.0 (12.8 to 15.0)	13.0 (11.0 to 14.0)	0.02*	-0.40
FMS-HS (score)	6.0 (5.0 to 6.0)	5.0 (5.0 to 6.0)	6.0 (5.0 to 6.3)	0.25	-0.21
FMS-BS (score)	5.0 (4.0 to 5.0)	4.0 (4.0 to 5.0)	4.0 (5.0 to 5.0)	0.47	-0.13
FMS-TS (score)	3.0 (2.0 to 4.0)	2.0 (2.0 to 3.5)	3.5 (2.0 to 4.0)	0.18	-0.24
PHE angle (°)	22.1 (10.6)	27.6 (9.1)	18.4 (10.0)	0.01*	0.43
ASLR angle (°)	76.2 (11.1)	75.0 (9.5)	77.0 (12.2)	0.60	0.09
Upper limb muscle mass (kg)	2.3 (0.7)	2.4 (0.7)	2.2 (0.7)	0.36	0.16
Lower limb muscle mass (kg)	7.4 (1.7)	7.8 (1.9)	7.2 (1.7)	0.34	0.17
Trunk muscle mass (kg)	20.0 (4.2)	20.9 (4.1)	19.5 (4.2)	0.35	0.16
SMI (kg/m^2^)	7.0 (1.0)	7.2 (1.0)	6.8 (1.0)	0.34	0.17

There were significant positive correlations between the FMS-FS and FMS-TS and upper/lower limb/trunk muscle mass and SMI in the LBP group (Table 4). FMS-HS was significantly positively correlated only with SMI (Table 4). The NLBP group showed significant positive correlations with FMS-TS, upper/lower limb/trunk muscle mass, and SMI, whereas FMS-BS and ASLR angles were significantly negatively correlated with upper/lower limb/trunk muscle mass and SMI (Table 5).

**Table 4:** Correlations between each measurement item in the lower back pain group * Significant correlation (p<0.05). PHE: prone hip extension; SMI: skeletal muscle mass index; FMS-FS: final score on the Functional Movement Screen; FMS-HS: higher-level movement pattern item score on the Functional Movement Screen; FMS-BS: basic mobility and stability movement pattern item scores on the Functional Movement Screen; FMS-TS: transitional movement pattern item score on the Functional Movement Screen; ASLR: active straight leg raise

Measurement item	Upper limb muscle mass		Lower limb muscle mass		Trunk muscle mass		SMI		PHE angle	
	Correlation coefficients	p-values	Correlation coefficients	p-values	Correlation coefficients	p-values	Correlation coefficients	p-values	Correlation coefficients	p-values
FMS-FS	0.59	0.03*	0.75	<0.01*	0.59	0.03*	0.76	<0.01*	0.24	0.40
FMS-HS	0.37	0.19	0.51	0.06	0.37	0.19	0.54	0.05*	0.15	0.61
FMS-BS	-0.40	0.16	-0.38	0.18	-0.43	0.12	-0.41	0.15	-0.19	0.51
FMS-TS	0.71	<0.01*	0.85	<0.01*	0.71	<0.01*	0.83	<0.01*	0.28	0.34
PHE angle	0.29	0.31	0.39	0.18	0.31	0.28	0.25	0.39	-	
ASLR angle	-0.42	0.14	-0.31	0.27	-0.45	0.11	-0.32	0.27	-0.32	0.27

**Table 5:** Correlation between each measurement item in the non-lower back pain group * Significant correlation (p<0.05). PHE: prone hip extension; SMI: skeletal muscle mass index; FMS-FS: final score on the Functional Movement Screen; FMS-HS: higher-level movement pattern item score on the Functional Movement Screen; FMS-BS: basic mobility and stability movement pattern item scores on the Functional Movement Screen; FMS-TS: transitional movement pattern item score on the Functional Movement Screen; ASLR: active straight leg raise

Measurement item	Upper limb muscle mass		Lower limb muscle mass		Trunk muscle mass		SMI		PHE angle	
	Correlation coefficients	p-values	Correlation coefficients	p-values	Correlation coefficients	p-values	Correlation coefficients	p-values	Correlation coefficients	p-values
FMS-FS	-0.11	0.64	-0.06	0.79	-0.10	0.67	-0.12	0.61	0.30	0.18
FMS-HS	0.06	0.81	0.03	0.90	0.06	0.80	0.06	0.79	0.10	0.66
FMS-BS	-0.54	0.01*	-0.44	0.05*	-0.52	0.02*	-0.58	<0.01*	0.21	0.35
FMS-TS	0.52	0.01*	0.50	0.02*	0.52	0.02*	0.53	0.01*	0.11	0.63
PHE angle	0.07	0.75	0.20	0.39	0.08	0.71	0.07	0.76	-	
ASLR angle	-0.45	0.04*	-0.46	0.04*	-0.44	0.05*	-0.48	0.03*	0.06	0.81

The FMS-FS (p=0.02, r=-0.40) and PHE angle (p=0.01, r=0.43) were significantly higher in the LBP group than in the NLBP group (Table 3).

Fourteen participants (100%) in the LBP group and 17 (81.0%) in the NLBP group had a left-right difference in one or more items, with no significant difference between the groups (p=0.08). Ten participants (71.4%) in the LBP group and 20 (95.2%) in the NLBP group had at least one item with a score of 1 or less, which was significantly higher in the NLBP group than in the LBP group (p=0.03).

**Figure 1:**
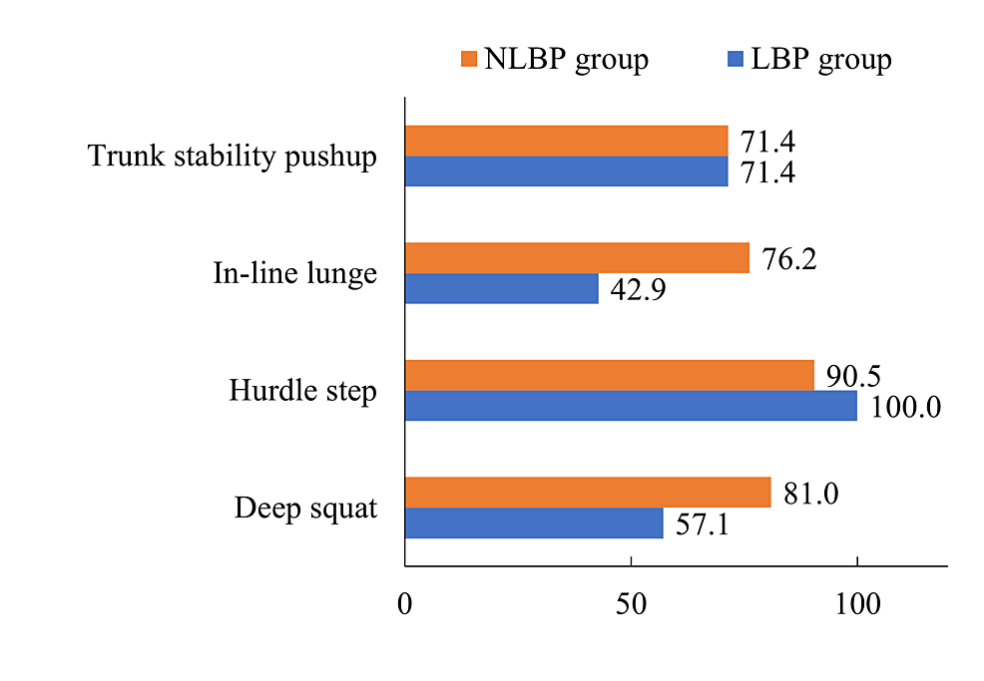
Percentage of participants who received a point reduction for each criterion on the Functional Movement Screen that included the word "spine" or "torso. NLBP: non-lower back pain; LBP: lower back pain

FMS items that included the word “spine” or “torso” in the measurement criteria were “deep squat,” “hurdle step,” “in-line lunge,” and “trunk stability pushup.” Figure 1 shows the percentages of participants who lost points based on these criteria. Only “in-line lunge” was significantly more common in the NLBP group than in the LBP group (p=0.05).

## Discussion

The purpose of this study was to clarify the relationship between FMS scores, including subcategories, skeletal muscle mass, and hip mobility, in office workers with or without chronic LBP, and to verify whether there are differences in each of the above items between office workers with or without chronic LBP.

The results showed that, in the NLBP group, the FMS-BS and ASLR angles were negatively correlated with skeletal muscle mass of the limbs and trunk and SMI, which are indices of skeletal muscle mass. Because joints tend to follow trajectories that cause less discomfort, such as tissue resistance and pain [10], it has been noted that movement patterns are influenced by other factors, such as tissue flexibility [23]. In particular, there is a positive correlation between muscle cross-sectional area and muscle size on CT and MRI, and the passive stiffness of the muscle [24,25]. Therefore, it is easy to understand why the FMS-BS, which easily reflects mobility, was negatively correlated with SMI in this study. The results of the present study also suggest that mobility decreases as muscle mass increases. Therefore, when training to increase muscle mass, it may be necessary to combine this with mobility training. In contrast, the LBP group showed no significant correlation between FMS-BS, ASLR angle, and skeletal muscle mass of the extremities and trunk, or SMI. This suggests that having LBP may result in a different relationship between mobility and muscle mass than the original relationship between mobility and muscle mass.

The FMS-TS was positively correlated with the skeletal muscle mass index in both the LBP and NLBP groups. “Trunk stability pushup” and “bird dog,” which are similar to “rotary stability,” especially require trunk muscle activity [26,27], and this task requires the control to move the limbs while keeping the trunk stable. Therefore, in the FMS-TS, larger muscle mass and easier access to muscle activity are advantageous for controlling the limbs and trunk.

Furthermore, the FMS-FS showed a significant correlation with the skeletal muscle mass index in the LBP group, but not in the NLBP group. Because the FMS-TS and FMS-BS contrasted in relation to muscle mass, the total score, FMS-FS, may result from the offsetting results of the FMS-TS and FMS-BS in the NLBP group. Therefore, it suggests that in participants without LBP, the FMS should not only focus on the final score but also the scores of the subcategories when evaluating them.

Regarding the LBP and NLBP groups, only the FMS-FS and PHE angles were significantly higher in the LBP group than in the NLBP group. The higher the FMS-FS score, the better the movement pattern [13,14,19]. Ko et al. [28] compared the FMS scores of participants with chronic LBP and healthy participants and reported that healthy participants had higher scores, which is different from the present results. One difference between the previous and present study is that several participants in the Ko et al. [28] study had items that scored 0 because pain existed while performing the FMS task, which may be related to the difference in the results from the present study. In the current study, no participants experienced pain during movement; therefore, pure movement patterns could be evaluated, which may have resulted in higher FMS-FS scores in the LBP participants than in the NLBP participants. In the FMS, trunk movement is a common evaluation criterion for many items, and smaller trunk movements are more likely to result in higher scores [19]. In the presence of pain, increased muscle activity and reduced motion have been reported because of fear-avoidance of pain associated with motion and protective mechanisms against tissue damage [20,29]. In this study, it is possible that the FMS scores were higher in participants with LBP because they chose movement patterns with less load on the lumbar region to avoid pain. This is consistent with the fact that the LBP group showed less trunk movement in the “in-line lunge.” However, the FMS-HS item, in particular, is a complex movement among the FMS items, and it is unclear whether lumbar movements were large. Therefore, the magnitude of the lumbar movement for each item needs to be investigated in future studies.

For injury prevention, a perspective different from that of the total score (FMS-FS) has been proposed. The FMS-FS scores higher by performing movements in a “perfect” movement pattern [19], but along with that, it has been pointed out the importance of looking at one point to determine if the person can move beyond the minimum standard and the presence of a left-right difference [13,19,30]. In the present study, the NLBP group had more items with a score of one than the LBP group, which is consistent with the FMS-FS results. Therefore, it can be inferred that the LBP group had better movement patterns than the NLBP group.

Furthermore, the LBP group had a significantly larger hip angle than the NLBP group with respect to the PHE. A previous study [20] reported that participants with LBP in PHE had greater motion in the hip than in the lumbar spine. The results of the PHE in this study are consistent with those of previous studies and the FMS results, and it is possible that the hip motion in the PHE was greater because of routine hip-dominant motion over the lumbar spine. This may explain the lack of correlation between the FMS-BS or ASLR angle and skeletal muscle mass only in the LBP group.

These results contradict the concept proposed in the kinesiopathological model, which states that inappropriate movement patterns can cause tissue damage [10]. However, it has been shown that those at high risk for LBP in the future have lower FMS scores [31], and those with LBP at the time of measurement and those at risk for LBP should be interpreted separately. It is possible that the LBP group in this study originally had a poor movement pattern, which caused LBP, and the pain subsequently changed the movement pattern in a positive direction. However, this remains unclear and requires verification in future studies.

Limitations

A limitation of this study is that the FMS did not include factors such as power, endurance, or change in direction [14]. Therefore, different results may be obtained in the evaluation of movement patterns with respect to elements missing in the FMS. In this study, the LBP group was not subgrouped by direction of motion, region (e.g., high and left-right differences), or tissue. It is recommended that LBP be subgrouped and characterized rather than considered as a single condition [32]. In this study, subgrouping may have produced different results, and further studies are needed. Furthermore, in this study, the assessment of muscle mass was made in rough body segments, such as the upper and lower limbs, and the relationship between the FMS and the size of each muscle could not be verified in detail. Similarly, mobility was evaluated only for active motion in the sagittal plane of the hip joint. Other directions of motion, passive motion, and mobility of other joints should be verified in the future.

## Conclusions

This study examined the relationship between FMS scores, including subcategories, skeletal muscle mass, and hip mobility, in office workers with or without chronic LBP, and whether there were differences in each of the above items between office workers with or without chronic LBP. The FMS should focus not only on the total score but also on the subcategory scores because the total and subcategory scores show different relationships with skeletal muscle mass and hip mobility. In addition, the relationship between office workers with or without LBP differed. Office workers with chronic LBP may have different movement patterns and greater hip extension angles than those without LBP.
